# The olive biophenol hydroxytyrosol in neutral aqueous solutions – a UPLC-MS/MS investigation of its stability and oxidative color formation

**DOI:** 10.3389/fnut.2025.1532087

**Published:** 2025-04-25

**Authors:** Yue Ling Wong, Samy Boulos, Laura Nyström

**Affiliations:** Institute of Food, Nutrition and Health, ETH Zurich, Zurich, Switzerland

**Keywords:** hydroxytyrosol, autoxidation, chromophore, color stability, tap water, olive pomace

## Abstract

Olive pomace is a by-product of the olive oil industry rich in biophenols such as hydroxytyrosol, with potential to be valorized as a functional ingredient. To investigate the stability of hydroxytyrosol in neutral aqueous solutions, the oxidative transformation of 50 ppm hydroxytyrosol in real and simulated tap waters over 5 days was studied with UV/Vis spectroscopy and UPLC–MS/MS. Simulated tap water systems consisted of 241 mg/L NaHCO_3,_ with or without 139 mg/L CaCl_2_, and a pH range of 6.5–8.0. Hydroxytyrosol in real tap water was completely transformed to various oxidation products after 1 day, exhibiting a marked red color (λ_max_ = 490 nm). Similarly, the simulated tap water system with a chemical composition closest to that of real tap water was largely able to reproduce this behavior with 85% hydroxytyrosol oxidation. A number of hydroxytyrosol oxidation products were annotated, including the red chromophore 2-(2-hydroxyethyl)-5-hydroxy-benzoquinone, which was formed through hydroxytyrosol autoxidation in the presence of bicarbonate ions. The chromophore’s formation without addition of transition metals, enzymes or H_2_O_2_ is presented for the first time. The results have implications for the stability and color behavior of olive pomace-derived biophenols applied to neutral or mildly alkaline environments.

## 1 Introduction

The consumption of olive oil has been steadily increasing in recent decades, which leads to growing amounts of olive mill waste generated. Olive pomace is the byproduct of the modern two-phase olive processing technique, with 6 million tons of pomace produced in Spain and an estimated 9–10 million tons produced in the European Union annually (1, 2). If left untreated, olive pomace is an environmental pollutant due to its high chemical oxygen demand and toxic level of polyphenols (3, 4). Up to 98% of phenolic compounds present in the olive fruit remains in the pomace after olive oil extraction, including tyrosol and hydroxytyrosol, as well as secoiridoids such as oleuropein and ligstroside (5). Hydroxytyrosol (HT) is often found to be the most abundant biophenol detected in olive pomace (6), due to hydrolysis and transformation of the secoiridoids during processing and storage steps. HT possesses high antioxidant properties, with cardioprotective, anticancer, neuroprotective and endocrine effects (7). Hence, olive pomace and its high content of biophenols such as HT has huge potential to be valorized for further use.

Given the demand for natural plant-derived ingredients in food and cosmetics instead of synthetic ones, olive pomace is a promising source of phenolics and antioxidants that can be valorized, harnessing the antioxidant and antimicrobial functionalities of the biophenols present. Biophenols from olive mill waste, with either a prior extraction step or the byproducts directly used, have since been applied to exploit their strong antioxidant and antimicrobial activities against foodborne pathogens in edible oils, grain products, fermented milk and in food packaging (8–12). For the success of such applications, maintaining the stability of these biophenols in the applied matrices such as in food or cosmetics and through any processing steps is crucial. As the food industry is likely to use tap water of varying composition in their production processes, it is important to analyze the stability or potential color changes of olive pomace extracts or biophenols in such a matrix. Additionally, it has been reported that thermal treatments on olive oils cause a significant decrease in HT- and tyrosol-like substances due to oxidation (13), and that losses in phenolic compounds like HT and in apple pomace occurred during heat treatment of functionalized extruded snacks and baked products (14, 15). As such, a detailed characterization and understanding of the stability of the main phenolic compounds is necessary for effective valorization of olive pomace and other byproducts.

Other than oxidation of olive biophenols by endogenous enzymes activated during various olive processing steps, nonenzymatic autoxidation can also occur across stages of processing, storage and application (16). Nonenzymatic autoxidation of phenolic compounds is influenced by factors including light, oxygen, pH, temperature and the presence of other chemicals such as metal ions. To date, studies on HT autoxidation include the identification of two main regioisomeric products from the autoxidation of HT in aqueous phosphate buffer at pH 7.4 (17) and the comparison of autoxidation products of various phenols in virgin olive oil to enzymatic and Fenton reaction oxidation products (16). It is known that the presence of metal ions not only affects compound stability but could also lead to color development of phenolic compounds, with the interaction of HT with ferric ions developing a blue color at pH 7.4 (18). It is not clear, however, if such color formation is caused by the chelation of the metal to HT without its breakdown, or if the color is due to oxidation products of the biophenol.

For efficient and optimal valorization of the olive pomace biophenols, their stability under various conditions relevant to, e.g., the food and cosmetic industries must be investigated. To this end, the observed spontaneous reaction in olive pomace biophenols leading to changes in absorption and color formation was explored. As part of preliminary stability tests, we have observed that an olive pomace extract dissolved in local tap water at a final concentration of 50 ppm HT developed a marked red color after 1 day, while the same extract in pure water showed no color development. The extract was obtained from Picual olive pomace, with the liquid phase of the pomace further purified to yield a concentrated, aqueous extract rich particularly in HT among other hydrophilic biophenols. To identify the most relevant biophenols responsible for the observed color developments, a preliminary study was set up including HT, oleuropein, verbascoside and 3,4-dihydroxyphenyl glycol. Among these biophenols, only HT showed a significant red color development similar to that of the olive pomace extract.

The aim of the present study was to investigate the link between HT stability and its color behavior. To this end, HT stability was evaluated in local tap water and simulated tap water systems of various composition (including bicarbonate and calcium ions) across a pH range of 6.5–8.0 by UV/Vis spectrophotometry and UPLC-MS/MS. The results revealed the synergistic roles of bicarbonate and calcium in the autoxidation of HT, with MS/MS annotation of colored oxidation products, and have implications for the use of HT and the valorization of olive pomace.

## 2 Materials and methods

### 2.1 Chemicals and materials

Hydroxytyrosol (HT) was purchased from Extrasynthese (Genay, France). Sodium bicarbonate, calcium chloride, manganese(II) sulfate monohydrate, copper(II) chloride and MOPS were purchased from Sigma Aldrich (St. Louis, MO, USA). Hydrochloric acid 37% was purchased from VWR Chemicals (Radnor, PA, USA). Formic acid, acetonitrile, methanol, 2-propanol, and water (all LC-MS grade) for UPLC-MS were purchased from Fisher Scientific AG (Reinach, Switzerland). Nickel(II) chloride hexahydrate was purchased from Riedel-de Haën (Seelze, Germany). Cobalt(II) chloride hexahydrate and zinc(II) sulfate heptahydrate were purchased from Fluka (Buchs, Switzerland). Water was purified using a Millipore Milli-Q system (Billerica, MA, USA). Tap water was obtained from the Laboratory of Food Biochemistry, ETH Zurich, Switzerland. The olive pomace extract used in preliminary stability tests was produced from Picual olive pomace and provided by the European project PHENOLIVA.

### 2.2 Preliminary tests on simulated tap water systems

The metal cations calcium, manganese, cobalt, nickel, copper and zinc were screened for the development of a simulated tap water system, by addition to 241 mg/L NaHCO_3_ ^1^(175 mg HCO_3_^−^) in Milli-Q water. The metal cations were added at their respective concentrations in local tap water, which was measured by inductively coupled plasma mass spectrometry ICP-MS (Table 1). The addition of 0.2 M MOPS solution adjusted to pH 7.0 and 7.5 to obtain a buffered simulated tap water system was also tested. To all these preliminary simulated tap water systems a final concentration of 50 ppm hydroxytyrosol (HT) was added and its transformation over 5 days was analyzed. Based on these tests, the final composition of our simulated tap water systems was determined.

**Table 1:** Concentration of metals ions in local tap water determined by inductively coupled plasma mass spectrometry (ICP-MS).

Metal ion	Local tap water	s.d.
Ca (mg/L)	50.0	0.2
Mn (μg/L)	1.34	0.02
Co (μg/L)	0.406	0.006
Ni (μg/L)	8.28	0.14
Cu (μg/L)	7.40	0.33
Zn (μg/L)	18.6	0.7

### 2.3 Oxidation of hydroxytyrosol in real and simulated tap water systems

Hydroxytyrosol (HT) was dissolved in local tap water to reach a final concentration of 50 ppm (TW). Simulated tap water systems with a final concentration of 50 ppm HT and 241 mg/L NaHCO_3_ (175 mg HCO_3_^−^) in Milli-Q water were constructed, with pH adjusted by HCl to obtain samples at pH 6.5, 7.0, 7.5 and 8.0 (pH 6.5, pH 7.0, pH 7.5, pH 8.0). Further simulated tap water systems were prepared with final concentrations of 50 ppm HT, 241 mg/L NaHCO_3_ and 139 mg/L CaCl_2_ (50 mg/L Ca), at pH 6.5, 7.0, 7.5 and 8.0 (pH 6.5 + Ca, pH 7.0 + Ca, pH 7.5 + Ca, pH 8.0 + Ca). As a control, HT was dissolved in Milli-Q water at a final concentration of 50 ppm (Control). All samples were prepared in triplicate, stored in the dark at room temperature and analyzed over 5 days.

### 2.4 Analysis of oxidation products and color development by UV/Vis spectroscopy and UPLC-ESI-qTOF-MS/MS

The development of red color of HT in the real and simulated tap water systems was analyzed over 5 days with a Cary 100 UV/Vis spectrophotometer, Agilent Technologies (Santa Clara, USA), in the wavelength range of 280–800 nm.

Ultraperformance liquid chromatography coupled to a UV/Vis photo diode array and mass spectrometer detectors (UPLC-PDA-MS) was used to follow the products during color formation of the aqueous HT solutions, with the method adapted from that of Tuncel & Yilmaz (19).

A Waters Acquity UPLC system with Acquity UPLC BEH C18 column (100 mm × 2.1 mm, 1.7 μm particle size) was used, coupled to an Acquity PDA detector, followed by a Synapt G2 quadrupole time-of-flight (qToF) analyzer with electrospray ionization (ESI) (Waters Corp., Milford, MA, USA).

Chromatographic conditions: Eluent A was composed of 0.1% formic acid in water, and eluent B was pure acetonitrile (ACN). The flow rate was 0.4 mL/min at 50°C, with a total run time of 24.1 min and an injection volume of 7.5 μL in PLNO mode (partial loop with needle overfill). The gradient started isocratically with 100% eluent A for 5 min, followed by a linear gradient to 80% A within 5 min, staying there for 1 min, going further down to 50% A within 6.3 min, then staying there for 0.9 min, to linearly go down to 0% A within 2.1 min, and staying there for 1.6 min, before switching back linearly to the initial conditions (100% A, 0% B) within 2.2 min. Strong wash solvent was a 3:1:1:1 mixture of water:methanol:acetonitrile:2-propanol, and the weak wash and seal wash were 10% ACN in water (all MS-grade).

Detector conditions: The PDA detector was set up with a UV-channel at 280 nm, to follow the amount of HT over time. Quantification of HT was based on a calibration curve (Supplementary Figure S1). The limits of detection (LOD) and quantification (LOQ) were calculated based on concentrations giving signal-to-noise ratios (S/N) of 3 and 10 respectively, where a LOD of 0.3 ppm and a LOQ of 1.0 ppm were obtained. The MS was calibrated with a 5 mM sodium formate solution (in 2-propanol/water 9:1). A leucine-enkephalin solution (2 ng/μL in 1:1 ACN/water + 0.1% formic acid) was used as the lockmass (*m*/*z* 554.2615), which was acquired every 120 s during the measurements (scan time of 0.3 s; correction applied with 5 scans averaged). The spectra were acquired in resolution mode and negative ionization mode. The capillary, sample cone, and extraction cone voltages were set at 2.5 kV, 30 V, and 4 V, respectively. The desolvation gas flow rate was 850 L/h at 450°C. The cone gas flow was 20 L/h and the source temperature was 150°C. Full scan mass spectra were acquired from *m*/*z* 50 to 1,200 with scan times of 0.4 s in centroid mode. Individual MS–MS spectra were also acquired under the same conditions, using a resolving quadrupole LM (low mass) resolution of 15 (instrument-specific setting that corresponds to a narrow 1 Da window to discriminate between co-eluting products of similar *m*/*z*), and with MS–MS functions of the specific *m*/*z* in question using appropriate collision energy ramps of 5–10 V, 10–20 V, and 10–30 V for parent ions of *m*/*z* < 250, *m*/*z* 250–350, and *m*/*z* > 350, respectively.

## 3 Results and discussion

Autoxidation of HT in tap water and simulated tap water systems was monitored over 5 days by UV/Vis spectrophotometry and UPLC-PDA-MS. Sodium bicarbonate was included in the simulated tap water systems to mimic the composition of real tap water, and the concentration of 241 mg/L NaHCO_3_ (175 mg HCO_3_^−^) was used as reported in a local water quality report. Calcium was also added in some of the simulated tap water systems at a final concentration of 50 mg/L, the concentration measured in local tap water by ICP-MS.

### 3.1 Preliminary tests to construct simulated tap water systems

An initial screening of other metal cations including manganese, cobalt, nickel, copper and zinc, at their respective concentrations in local tap water measured by ICP-MS, as additions to our simulated tap water systems with bicarbonate was performed. However, the addition of these metals showed no individual or additive effect on HT autoxidation and the concurrent red color development. As such, the final composition of our simulated tap water systems consisted of bicarbonate with and without calcium, adjusted to pH 6.5, 7.0, 7.5 and 8.0 to cover the pH range reflective of tap waters. HCl was chosen to adjust the pH of the simulated tap water systems over other buffer systems such as phosphate buffers which are known to interact with metal ions like Ca^2+^ (20), or MOPS which when tested was found to cause a different HT oxidation pathway, with no color development.

### 3.2 Color development of hydroxytyrosol in real and simulated tap water systems

**Figure 1:**
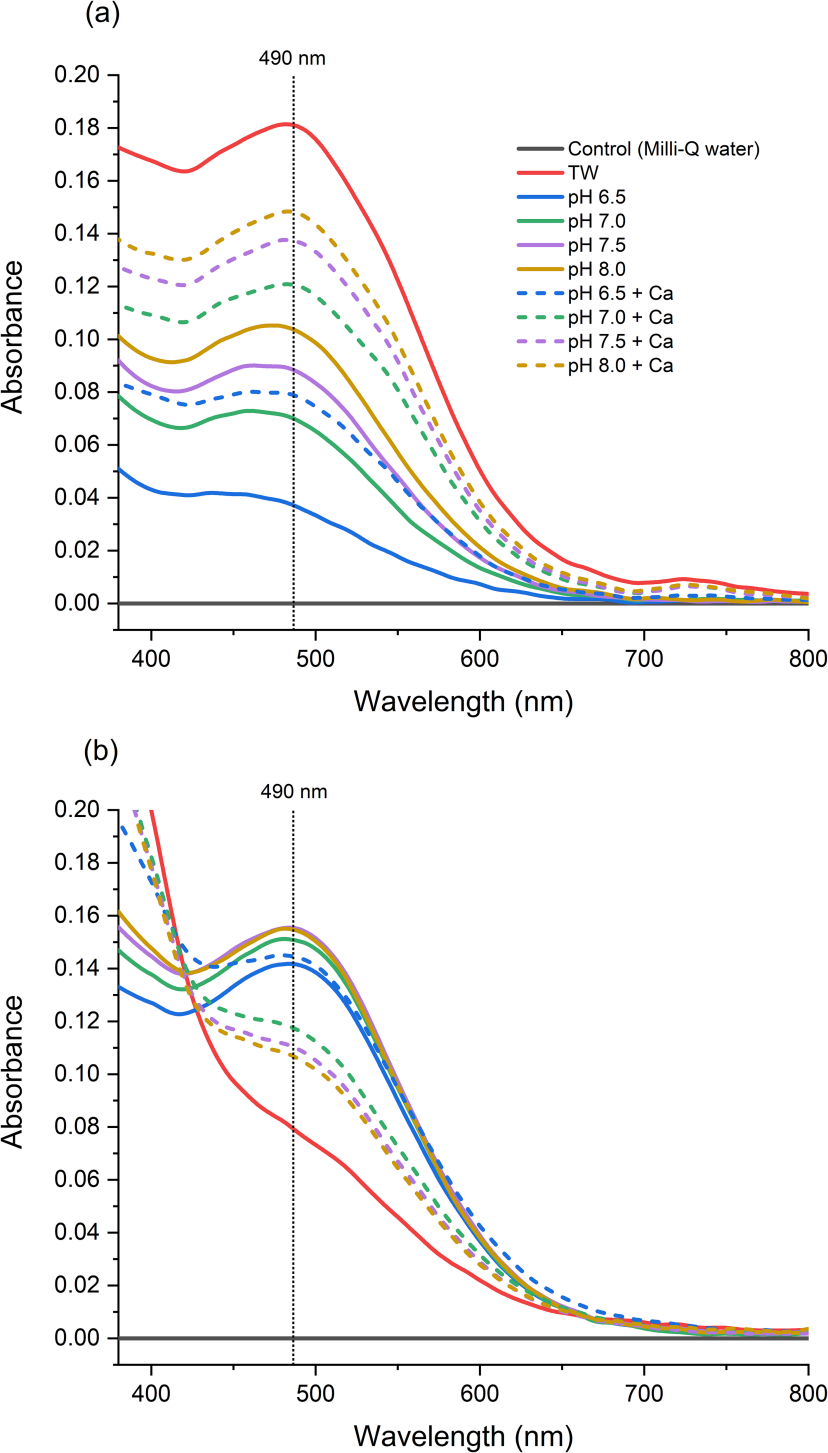
Optical spectra of 50 ppm hydroxytyrosol (HT) solutions **(a)** after 1 day; **(b)** after 5 days at room temperature in the dark. The samples are HT solutions in: Milli-Q water (Control), local tap water (TW), pH-adjusted simulated tap waters with 175 mg/L bicarbonate (pH 6.5, pH 7.0, pH 7.5, pH 8.0) and pH-adjusted simulated tap waters with 175 mg/L bicarbonate and 50 mg/L calcium (pH 6.5 + Ca, pH 7.0 + Ca, pH 7.5 + Ca, pH 8.0 + Ca).

Solutions of 50 ppm HT in Milli-Q water (Control) showed no absorption in the visible wavelength range during storage (Figure 1a). In contrast, solutions of 50 ppm HT in local tap water (TW) exhibited a marked red color after 1 day (Supplementary Figure S2), with a peak absorbance of A = 0.18 at λ_max_ = 490 nm (Figure 1a). Solutions of 50 ppm HT in all simulated tap water systems also showed a red color of varying intensity after 1 day. A more intense red color development in all the simulated tap water systems with calcium was observed, and in the systems both with and without calcium a more intense red color development with increasing pH was observed (Figure 1a). The simulated tap water system adjusted to pH 7.5, closest to the pH 7.7 of local tap water, had A = 0.09 at *λ* = 490 nm, with the addition of calcium to the system causing an increase by 55% to A = 0.14. The simulated tap water system with bicarbonate but without the addition of calcium was not sufficient in mimicking actual tap water, with the system at pH 8.0 (pH 8.0) displaying a red color with an absorbance only 55% that of actual tap water (Figure 1a). The addition of calcium better simulated tap water conditions, with the red color of the solutions at pH 8.0 (pH 8.0 + Ca) reaching an absorbance 83% that of actual tap water. Hence, the addition of 241 mg/L NaHCO_3_ and 50 mg/L Ca come close to, while not fully simulating actual local tap water conditions. Other factors such as a synergistic effect of other metals or complex anion systems present in tap water may explain the observed faster and stronger color development of HT in tap water. Nevertheless, the results account for the general trend to explain the responsible main actors for color development: HT transformed through the presence of bicarbonate and calcium, increasing in intensity with increasing pH in the range 6.5–8.0.

After storage for 5 days, the solutions of 50 ppm HT in Milli-Q water (Control) still showed no absorption in the visible wavelength range (Figure 1b). The solutions of 50 ppm HT in local tap water (TW) that showed the stronger red color after 1 day, had the lowest absorbance after 5 days and faded to a yellow color, as indicated by the drop in absorbance at *λ* = 490 nm to A = 0.08. A similar trend of a decrease in red color and absorbance at λ = 490 nm was also observed in the solutions of simulated tap waters with calcium after 5 days, for instance from A = 0.15 (day 1) to 0.11 (day 5) for the pH 8.0 + Ca sample. In contrast, the solutions of simulated tap waters without calcium showed an increase in red color development after 5 days, with absorbance at λ = 490 nm reaching A = 0.15 (pH 7.5, pH 8.0). The effects of calcium and pH in the simulated tap water systems are further discussed in Section 3.3. Furthermore, when acidifying the red solutions, we observed a loss of absorbance at 490 nm leading to a color change to light yellow.

### 3.3 Products of hydroxytyrosol autoxidation and link to color development

**Figure 2:**
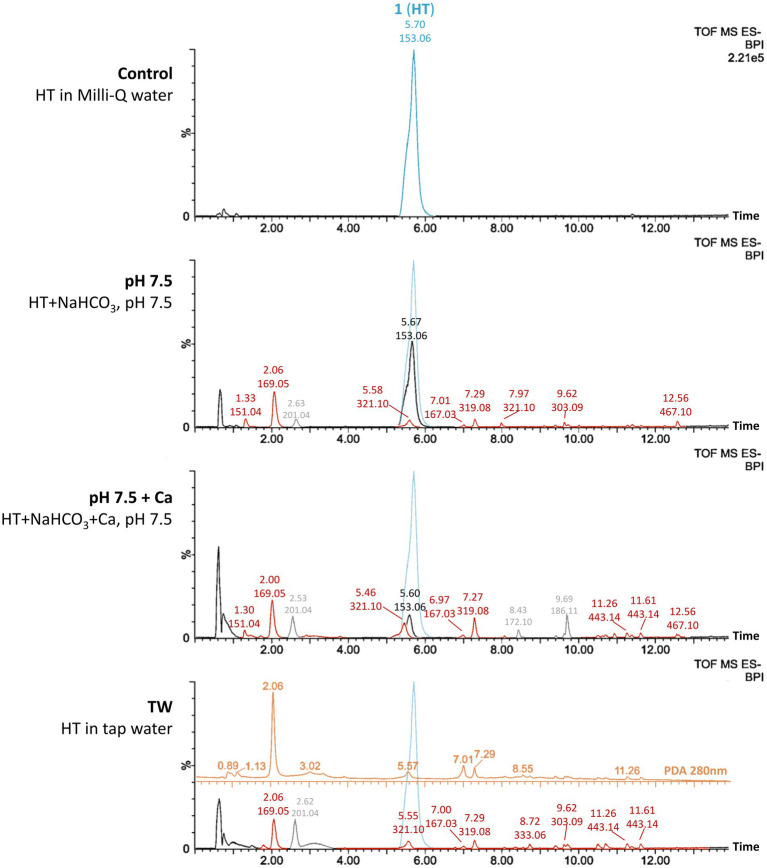
Base peak ion (BPI) chromatograms from UPLC-MS analysis in negative mode of 50 ppm hydroxytyrosol (HT) solutions in Milli-Q water (Control), local tap water (TW), pH-adjusted simulated tap waters with 175 mg bicarbonate (pH 7.5) and pH-adjusted simulated tap waters with 175 mg bicarbonate and 50 mg/L calcium (pH 7.5 + Ca) after 1 day at room temperature in the dark. (Samples pH 7.5 and pH 7.5 + Ca are representative in terms of profile of detected species for the simulated tap water systems across the pH range 6.5–8.0 without and with calcium). Peaks are labeled with the retention time (min) and the corresponding base peak m/z. All y-axes are scaled like the top chromatogram (Control), showing the original peak of the starting material HT (m/z 153.06) overlayed as light blue peak in each chromatogram for relative comparison. The signal of m/z 321.10 coeluting with HT was made visible in the BPI chromatograms by overlaying the extracted ion chromatogram for this species (extraction window:0.025 Da). Peaks annotated in gray color are contaminants that are not UV-active at 280 nm and hence not related to HT products and not further discussed. For the TW sample, the PDA spectrum at 280 nm is further overlayed above the BPI chromatogram in orange color, with its y-axis depicting arbitrary units.

To elucidate the process of color formation of the HT solutions, the various solutions were analyzed with a reversed-phase UPLC-PDA-MS/MS system running in negative mode. As chromatographic separation required acidic conditions, we relied on the MS detection after UPLC separation, as the analytes in their protonated form do not absorb at 490 nm (see Supplementary Figure S3). While the 50 ppm HT solutions in Milli-Q water (Control) predictably only showed the HT starting material (*m*/*z* 153.06 at 5.7 min), all other samples showed additional peaks to varying degrees (Figure 2). Looking at the amount of HT present in the simulated tap water systems relative to the starting amount in the Control, there is a clear decreasing trend as bicarbonate and calcium ions are involved. After 1 day, 60% of HT was transformed in the simulated tap water system adjusted to pH 7.5 (pH 7.5). In the simulated tap water at the same pH 7.5 with the addition of calcium, this increased to 85% HT transformed (pH 7.5 + Ca), compared to in the real tap water (TW) where HT was no longer detectable. Across the pH range of 6.5–8.0 in the simulated tap waters, the amount of HT remaining also decreased the higher the pH. After 1 day, 66% of HT remained intact in the simulated tap water system adjusted to pH 6.5 (pH 6.5), with 50, 42 and 36% for the pH 7.0, pH 7.5 and pH 8.0 samples, respectively. The fact that the degree of transformation of HT correlates with the red color formation, with the strongest colored sample (TW) having no residual HT at all, strongly indicates that the mechanism of color formation is not purely chelation-based (i.e., colored HT-metal complexes), but is rather linked to products formed from the transformation of HT.

The presence of calcium, as well as an increasing pH in the simulated tap water systems led to a larger extent of HT degradation with a corresponding greater intensity of the red color development. The presence of metal cations like calcium is known to exert a catalytic effect on the oxidation of phenolic compounds like HT (21, 22), due to the increased deprotonation of the catecholic hydroxyl groups leading to increased susceptibility of dioxygen-induced oxidation (23). Similarly, an alkaline pH has been shown to negatively affect the stability of phenolic compounds such as HT, attributed to the deprotonation of the phenolic hydroxyl groups (23–26).

**Figure 3:**
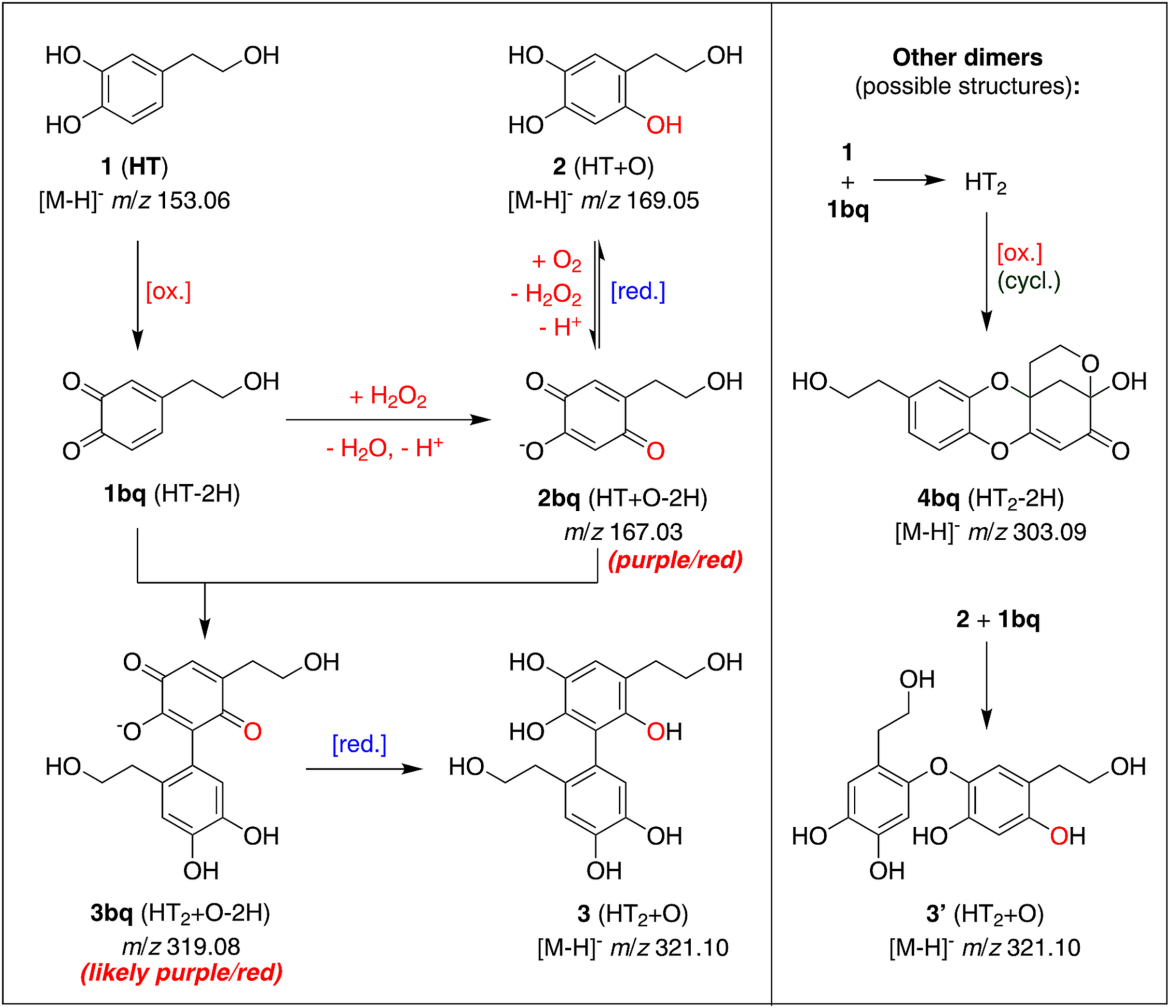
Suggested chemical structures of the main autoxidation products of hydroxytyrosol (HT) in real and simulated tap waters, based on the UPLC-MS/MS analysis and literature precedence. Structures are labeled with numbers in their reduced form (e.g., 1 = HT) and are followed by “bq” for their respective benzoquinone form (e.g., 1bq = HT-2H).

The profile of formed products from HT transformation was similar for all simulated and real tap water samples across the pH range 6.5–8.0 (Figure 2). The corresponding detected species are oxidation products of HT and are systematically listed in Table 2. Apart from uptake of oxygen atoms and loss of 2H, oxidative dimerization was observed with HT_2_-/HT_3_-based species. The three main products observed in all HT solutions were *m*/*z* 169.05, *m*/*z* 321.10 and *m*/*z* 319.08, the former with an additional oxygen (HT + O) and the latter two are dimers with an additional oxygen (HT_2_ + O and HT_2_ + O - 2H, respectively). Based on the literature, mechanistic considerations with regard to the oxidative dimerization, and the MS/MS spectra, we propose possible structures for some of the observed oxidation products (Figure 3).

**Table 2:** List of detected species, their observed retention time (R.T.), molecular ion [M-H]^−^, and MS/MS fragmentation patterns using RP-UPLC-MS/MS for the analysis of autoxidation of 50 ppm HT solutions in real and simulated tap waters.^a^

Class	#	Species	Chemical formula	[M – H]^−^ (*m*/*z*)	R.T. (min)	MS/MS fragments^b^ (*m*/*z*)
Monomers	1	HT^c^	C_8_H_10_O_3_	153.06	5.7	123.04; 122.04
		HT – 2H	C_8_H_8_O_3_	151.04^d^	1.3	151.04; 123.04; 122.04
	2	HT + O	C_8_H_10_O_4_	169.05	2.0	151.04; 139.04; 124.02; 121.03
	2bq	HT + O – 2H	C_8_H_8_O_4_	167.03	7.0	149.02; 137.02; 121.03; 109.03
Dimers		HT_2_	C_16_H_18_O_6_	(305.10)	*n.o.* ^e^	-
	4bq	HT_2_ – 2H	C_16_H_16_O_6_	303.09	9.6	273.08; 245.08; 229.06
	3	HT_2_ + O	C_16_H_18_O_7_	321.10	5.5	303.09; 291.09; 255.07; 246.05
	3’				8.0	303.09; 273.08; 258.05; 243.06
	3bq	HT_2_ + O – 2H	C_16_H_16_O_7_	319.08	7.3	301.07; 289.07; 253.05; 244.04; 241.05
Trimers		HT_3_	C_24_H_26_O_9_	(457.15)	*n.o.* ^e^	-
		HT_3_ + O – CH_2_O	C_23_H_24_O_9_	443.14	11.3	247.10; 217.09; 153.08
					11.6	247.10; 217.09; 153.04
		HT_3_ + O – 6H	C_24_H_20_O_10_	467.10	12.6	314.04; 258.05

a The PDA spectra from 210 to 498 nm of the detected species are presented in Supplementary Figure S3.

b Main fragment ions of collision induced fragmentation (top max. 5 fragment ions observed).

c Starting material hydroxytyrosol (HT).

d 3,4-Dihydroxyphenylglycol (as compared by a standard), with extensive in-source fragmentation under loss of H_2_O, such that it shows up mainly as *m*/*z* 151.04 in the BPI chromatogram (instead of its [M-H]^−^ of *m*/*z* 169.05).

e Not observed.

Under conditions of tyrosinase oxidation of 1 mM HT at pH 7.4 in a phosphate buffer, Vogna et al. (17) observed, isolated and fully characterized methano-oxocinobenzodioxinone (4bq) as the main product. The authors mentioned that the autoxidation of HT without enzyme in phosphate buffer at pH 7.4 also produced the same main detectable product after 2 days. Hence, our observed *m*/*z* 303.09 species likely has the same structure. For possible structures of our main products of interest, *m*/*z* 169.05, *m*/*z* 319.08 and 321.10, the literature is more divided. Roche et al. (27) observed next to *m*/*z* 303 and 167 also a species of *m*/*z* 319 (with *m*/*z* 289 and 241 as fragments) in the oxidation of HT using the free-radical generating azo-compound AAPH at 37°C and pH 7.4 in phosphate buffer, in agreement to our *m*/*z* 319.08 species at 7.3 min. The authors proposed a structure with C-C-linked dimers connected over the benzylic carbon of HT (Ar-CH_2_-CH_2_-OH; Ar = aromatic ring), based on mechanistic considerations, the chromatographic retention time, and the fact that the MS/MS spectrum exhibited no fragments corresponding to HT monomers, ruling out C-O-linkage, e.g., via aryl ether formation. On the other hand, De Lucia et al. (28) isolated and fully characterized products, after reduction by NaBH_4_ and peracetylation, that correspond to structures 2 (*m/z 169.05*) and 3 (*m/z 321.10*) in Figure 3, albeit originating from horseradish peroxidase/H_2_O_2_ oxidation of HT at pH 7.4 in a phosphate buffer. With rapid consumption of the HT substrate also came the development of a red coloration with an absorption maximum at 490 nm, identical to our observations. Given a closer similarity of HT oxidation systems as well as the comparable color development, our HT oxidation products were tentatively assigned considering the product structures presented by De Lucia et al. (28).

According to De Lucia et al., the role of the peroxidase enzyme was to produce the ortho-benzoquinone of HT (1bq), which can be formed as well by sodium periodate or autoxidation. The next step involves H_2_O_2_ to produce 2-(2-hydroxyethyl)-5-hydroxy-benzoquinone, in its deprotonated form 2bq (*m*/*z* 167.03), which is according to the authors the hydroxyquinone species responsible for the red coloration developed with a λ_max_ at 490 nm. In agreement with this is the observation of Wehrli et al. (29), showing the formation of a red chromophore with λ_max_ = 495 nm following the autoxidation of 6-hydroxydopamine, a molecule with the same aromatic core as dihydroxytyrosol 2, under aqueous conditions at pH 7. Dimerization by reaction of the chromophore 2bq with 1bq then leads to one of our main autoxidation products 3bq of *m*/*z* 319.08 (Figure 3), sharing the same hydroxyquinone core. Its reduced form 3 with *m*/*z* 321.10 was also observed, tentatively assigned to the peak coeluting with HT (R.T. = 5.5 min), as well as an isomer 3′ at 8.0 min, which due to the higher retention time, is possibly an aryl-ether dimer. Steric hindrance in the biphenyl 3bq would prevent planarity of the molecule (30), hence the conjugation and chromophore properties of 2bq would remain similar in 3bq, also likely contributing to the red color development we observed, which correlates to the absorbance at 490 nm.

**Figure 4:**
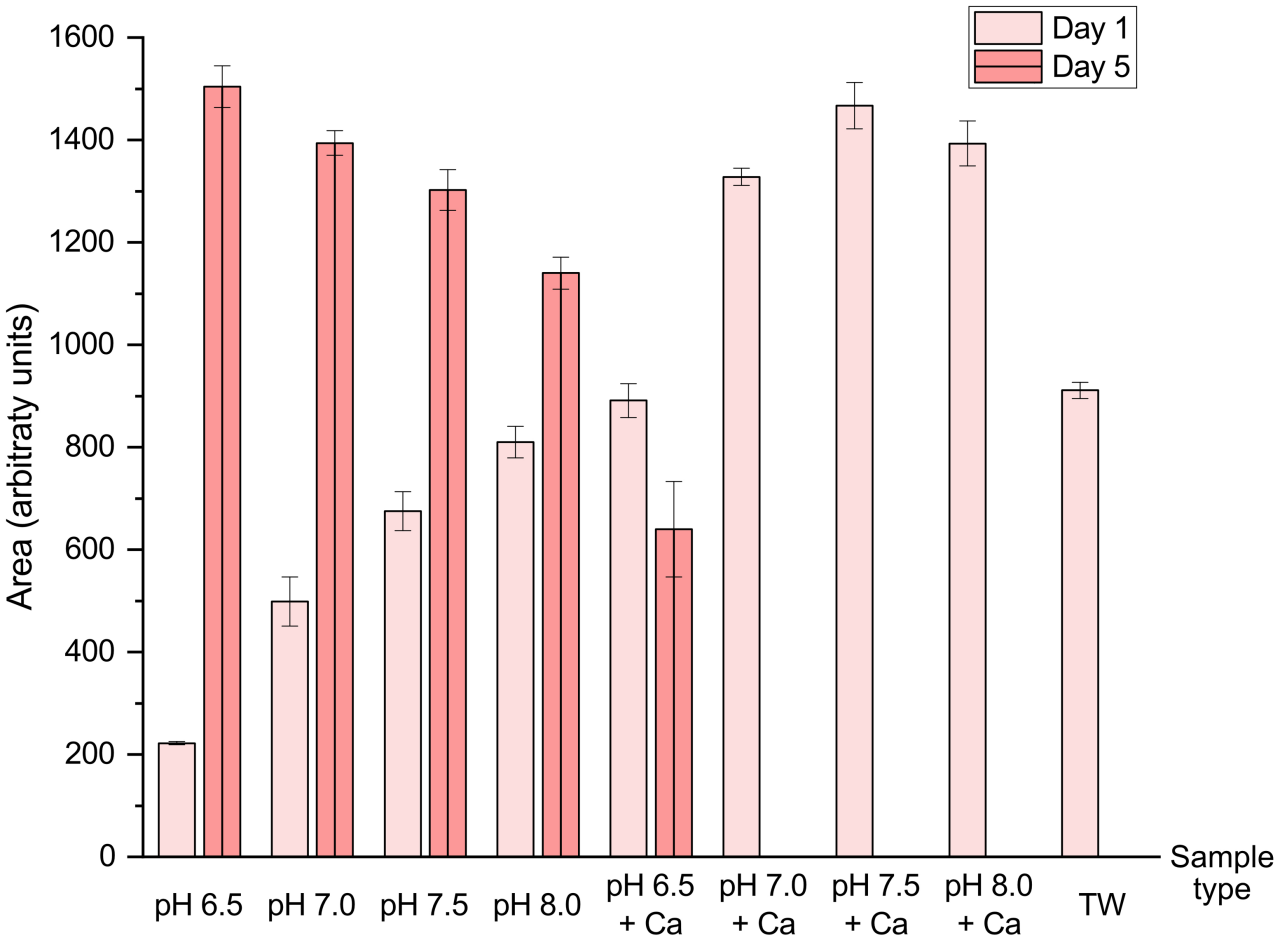
Summed peak areas of the proposed red chromophore species m/z 167.03 (2bq) and m/z 319.08 (3bq) at 280 nm using RP-UPLC-MS/MS for the analysis of autoxidation of 50 ppm hydroxytyrosol solutions in real and simulated tap waters with storage over 1 or 5 days. The average of triplicates and the respective standard deviations are reported.

Although an accurate quantification of the red chromophores 2bq and 3bq could not be performed due to the lack of commercial standards, the UPLC-PDA-MS measurements allowed for the sum of their peak areas at 280 nm to be calculated and to depict the general trends in the observed color formation across the different real and simulated tap water systems over storage for 1 or 5 days (Figure 4). For simplicity, here the extinction coefficients of 2bq and 3bq at 280 nm are approximated to be the same. The 2bq and 3bq species could be observed in all samples other than in HT in Milli-Q water (Control) sample, and after storage for 5 days in both the real tap water (TW) and the simulated tap water systems with the addition of calcium from pH 7.0–8.0 (pH 7.0 + Ca, pH 7.5 + Ca, pH 8.0 + Ca). The amount of the 2bq and 3bq species reflect a similar trend of the red color development across simulated tap water samples as measured by increased absorbance at 490 nm (Figure 1) with the addition of calcium, and also with a higher pH in the range 6.5–8.0. Also in agreement with the UV/Vis absorbance data is the increase in amount of 2bq and 3bq from 1 to 5 days of storage in the simulated tap waters without calcium. However, the summed amount of 2bq and 3bq in the real tap water (TW) after 1 day of storage is about a third less than that of the simulated tap water system with the most similar properties to real tap water (pH 7.5 + Ca), even though the real tap water (TW) samples showed the highest absorbance at 490 nm and the most intense red color, indicating additional compounds responsible for the red color development in the HT autoxidation process in real tap water conditions. The red chromophores 2bq and 3bq are present and key contributors to the overall absorbance at 490 nm in the earlier stages of HT autoxidation, and likely react further to larger polymeric compounds as similarly found in the enzymatic oxidation of HT by Xie et al. (31). The larger polymers would retain a similar chromophore core and red color but were not detectable by the present experimental conditions. The catalytic effect of HT oxidation in the presence of calcium was reflected by the lack of detection of the 2bq and 3bq species after 5 days of storage in the real tap water (TW) and the simulated tap water systems with the addition of calcium from pH 7.0–8.0 (pH 7.0 + Ca, pH 7.5 + Ca, pH 8.0 + Ca), indicating a faster rate of oxidation of HT in these systems, with the colored species further reacting to other products.

For the production of 2bq and 3bq, H_2_O_2_ is reportedly crucial, and is present in the De Lucia et al. (28) reaction system of peroxidase/H_2_O_2_. No hydrogen peroxide was added to our HT solutions in the simulated tap waters, yet we observed the same red color and products. However, there is literature evidence that the production of reactive oxygen species is linked to the presence of bicarbonate ions (32), which play a pro-oxidative role known to happen during the autoxidation of adrenaline, an analog of HT. This might explain why the production of chromophore 2bq is observed in the autoxidation of HT in simulated tap water in the presence of bicarbonate, namely due to the generation of oxidizing agents from the dissolved oxygen. Other oxidation steps might also produce H_2_O_2_ as a side reaction, as reported by Wehrli et al. (29), who observed as mentioned above in the autoxidation of 6-hydroxydopamine (an analog of dihydroxytyrosol 2) the formation of a red/purple chromophore under production of H_2_O_2_ in neutral solutions. Additionally, the red/purple chromophore was observed by the authors to turn yellow when protonated. Indeed, as mentioned above, when we acidified our red solutions after autoxidation of HT in tap water, we observed the same color change due to the protonation of 2bq, which in neutral solutions is in its conjugated base form due to its high acidity as a vinylogous carboxylic acid.

The reactions shown in Figure 3 are proposed to be the process of HT autoxidation and linked color formation in the presence of bicarbonate ions, shown for the first time to take place without the addition of transition metals, enzymes and/or H_2_O_2_. The main factor responsible for the color formation of HT at neutral pH does not seem to be the metal cations in tap water but instead autoxidation in the presence of bicarbonate, which produces the red chromophores. However, the presence of calcium at tap water concentrations, which usually is not redox active, intensifies the autoxidation of HT and the concurrent chromophore production, either by activating HT via chelation or by being involved in the reactive oxygen species production.

The results of this work have implications for the use of HT as a functional ingredient in food, and the sustainable valorization of millions of tons of waste streams like olive pomace that are rich in biophenols. Till now, HT has been tested as a functional food ingredient across product categories including edible oils, beverages, bakery, dairy, and other vegetable- and animal-based products (33). As HT is usually applied to food to exploit its antioxidant properties for better product stability, its own stability –or lack thereof– in the presence of bicarbonate, metal cations like calcium, and mild alkaline aqueous environments call for careful testing of its efficacy as a natural antioxidant additive in a given food matrix or after a processing step. Future work should explore the stability of HT and similar plant biophenols or extracts as natural additives in different food and cosmetic applications, as well as approaches to stabilize such compounds for more effective utilization.

## 4 Conclusion

This work demonstrated the autoxidation of hydroxytyrosol (HT) with concurrent red color development, under real and simulated tap water systems across a pH range of 6.5–8.0. Color formation of HT was monitored with UV/Vis spectroscopy and UPLC-MS/MS over 5 days, showing a larger extent of HT transformation and formation of autoxidation products with increasing pH. The structures of the main oxidation products were proposed based on the UPLC-MS/MS data and literature precedence, including the red chromophore 2-(2-hydroxyethyl)-5-hydroxy-benzoquinone and its dimer which are likely key contributors to the observed red color formation. This process of HT oxidation in the presence of bicarbonate ions, but without added transition metals, enzymes and/or H_2_O_2_, was presented in detail for the first time. The presence of calcium at concentrations found in tap water in the simulated tap water systems was also shown to catalyze a faster HT oxidation and a stronger color formation. The results of this work highlight the need to determine the stability and efficacy of valorized biophenols from waste streams in their applied food matrices. To avoid a decrease in stability or the transformation of compounds like HT, as well as unwanted color changes which may affect the visual properties of the final product, important factors such as pH, presence of metal cations and anions such as bicarbonate have to be controlled.

## Data Availability

The original contributions presented in the study are included in the article/Supplementary material, further inquiries can be directed to the corresponding author.

## Footnotes

^1^Value derived from the report “Die Qualität des Zürcher Trinkwassers” from the Stadt Zürich Wasserversorgung, WVZ/Q/2022.
