# Supplementary material for: The olive biophenol hydroxytyrosol in neutral aqueous solutions – a UPLC-MS/MS investigation of its stability and oxidative color formation
Source: Front Nutr. 2025 Apr 25;12:1532087. doi: 10.3389/fnut.2025.1532087 (PMC12061927; doi:10.3389/fnut.2025.1532087)
Supplement: Supplementary file 1 [file Table_1.docx]

Supplementary Information

**The olive biophenol hydroxytyrosol in neutral aqueous solutions – a UPLC-MS/MS investigation of its stability and oxidative color formation**

**Yue Ling Wong, Samy Boulos, Laura Nyström^*^**

Laboratory of Food Biochemistry, Institute of Food, Nutrition and Health, Department of Health Sciences and Technology, ETH Zurich, Zurich, Switzerland

*** Correspondence:**Laura Nyström
[laura.nystroem@hest.ethz.ch](mailto:laura.nystroem@hest.ethz.ch)


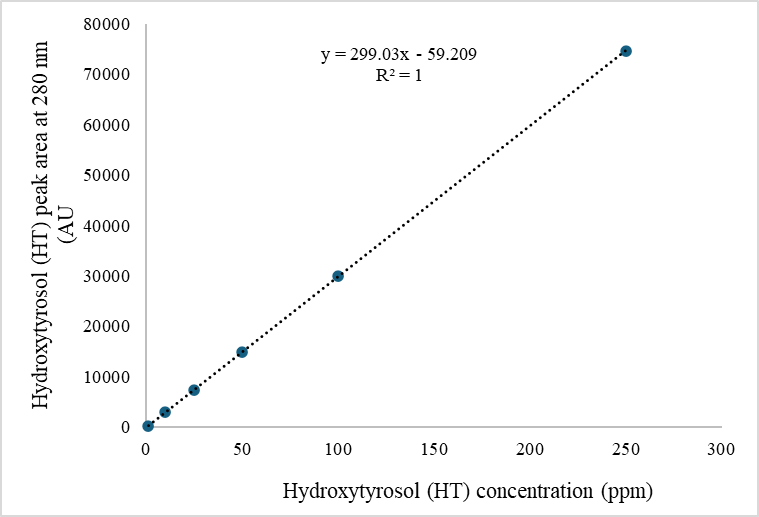


**Supplementary Figure S1**: Calibration curve of hydroxytyrosol (HT) from the concentration range 0, 10, 25, 50, 100 and 250 ppm.


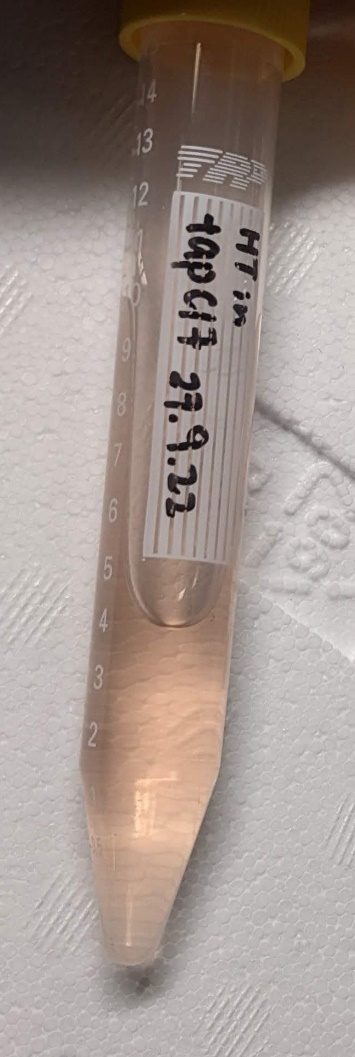


**Supplementary Figure S2**: Photo of the observed red color of 50 ppm hydroxytyrosol (HT) in local tap water after 1 day.


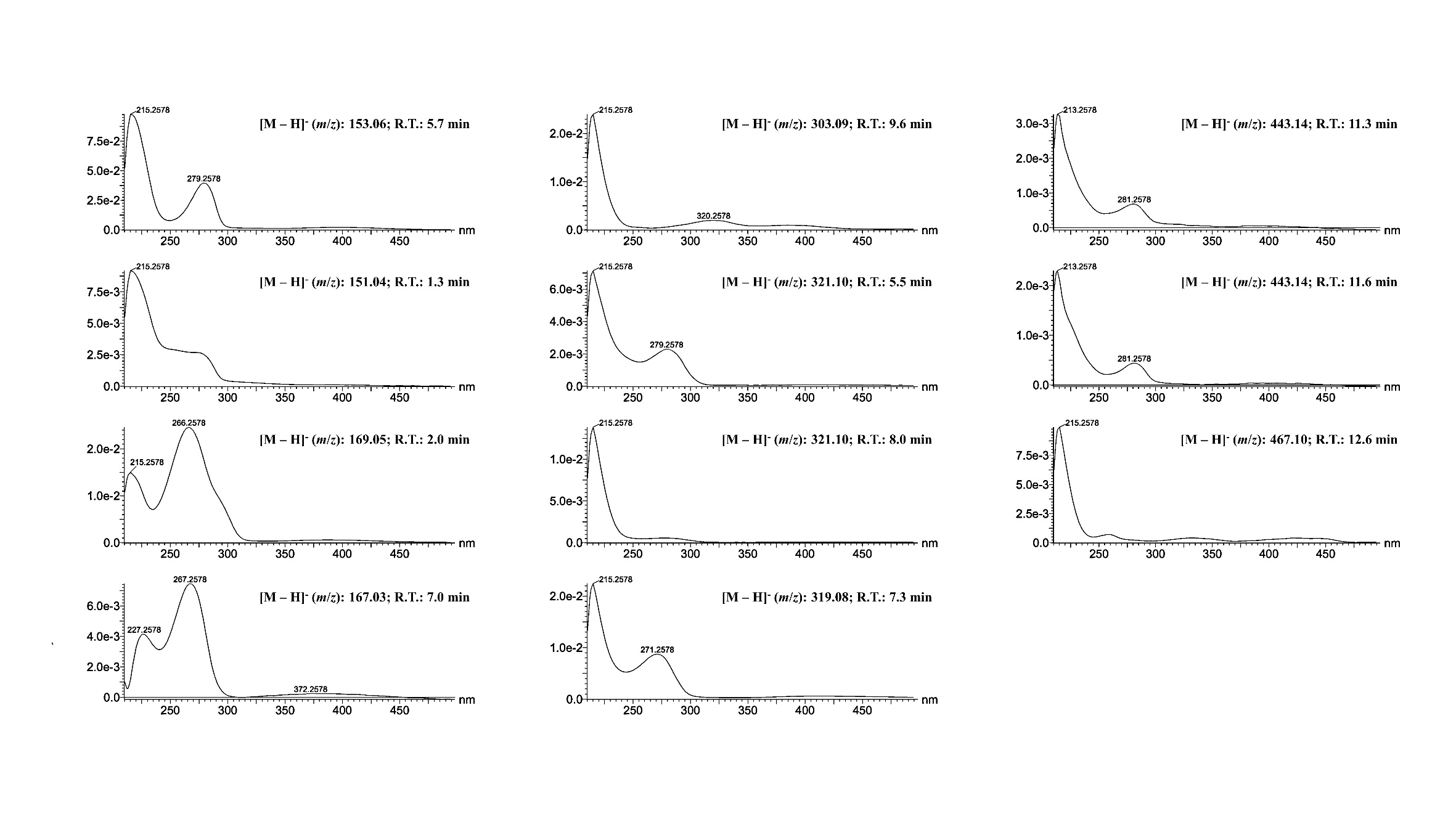


**Supplementary Figure S3**: DAD spectra from 210-498 nm of the detected species listed in **Table 2**, autoxidation products of HT. The best quality spectra of each species were taken from different samples as representatives. All y-axes depict arbitrary units. As the UPLC-PDA-MS analysis was performed under acidic conditions, these species were hence protonated, showing different absorbance at 490 nm as compared to the data obtained from the UV-Vis spectrophotometer, displayed in **Figure 1**.
